# An Explainable Statistical Method for Seizure Prediction Using Brain Functional Connectivity from EEG

**DOI:** 10.1155/2022/2183562

**Published:** 2022-12-08

**Authors:** Hao Chen, Taoyun Ji, Xiang Zhan, Xiaoxin Liu, Guojing Yu, Wen Wang, Yuwu Jiang, Xiao-Hua Zhou

**Affiliations:** ^1^Beijing International Center for Mathematical Research, Peking University, No. 5 Yiheyuan Road, Haidian District, Beijing 100871, China; ^2^Department of Pediatrics and Pediatric Epilepsy Center, Peking University First Hospital, No. 1 Xi'an Men Street, West District, Beijing 100034, China; ^3^Department of Biostatistics, School of Public Health, Peking University, No. 38 Xueyuan Road, Haidian District, Beijing 100083, China; ^4^Pazhou Lab, Guangzhou 510330, Guangdong, China

## Abstract

**Background:**

Epilepsy is a group of chronic neurological disorders characterized by recurrent and abrupt seizures. The accurate prediction of seizures can reduce the burdens of this disorder. Now, existing studies use brain network features to classify patients' preictal or interictal states, enabling seizure prediction. However, most predicting methods are based on deep learning techniques, which have weak interpretability and high computational complexity. To address these issues, in this study, we proposed a novel two-stage statistical method that is interpretable and easy to compute.

**Methods:**

We used two datasets to evaluate the performance of the proposed method, including the well-known public dataset CHB-MIT. In the first stage, we estimated the dynamic brain functional connectivity network for each epoch. Then, in the second stage, we used the derived network predictor for seizure prediction.

**Results:**

We illustrated the results of our method in seizure prediction in two datasets separately. For the FH-PKU dataset, our approach achieved an AUC value of 0.963, a prediction sensitivity of 93.1%, and a false discovery rate of 7.7%. For the CHB-MIT dataset, our approach achieved an AUC value of 0.940, a prediction sensitivity of 93.0%, and a false discovery rate of 11.1%, outperforming existing state-of-the-art methods. *Significance*. This study proposed an explainable statistical method, which can estimate the brain network using the scalp EEG method and use the net-work predictor to predict epileptic seizures. *Availability and Implementation*. R Source code is available at https://github.com/HaoChen1994/Seizure-Prediction.

## 1. Introduction

Epilepsy is a group of chronic neurological disorders characterized by the abnormal and excessive firing of brain neurons, called epileptic seizures [1]. According to the newest WHO global report on epilepsy, around 50 million people are suffering from epilepsy globally [2, 3]. During epileptic seizures, electrical activities in the brain are disrupted, resulting in dysfunction and communication disorders among brain regions, which in turn lead to many temporary symptoms, such as loss of consciousness, staring, and disturbances of movement [4]. Unpredictable seizures dramatically affect the life of patients and may even lead to death [5]. Therefore, accurate and reliable seizure prediction can be beneficial for treating epilepsy. Patients can use Anti-Seizure Medications (ASMs) for treatment in advance, which would substantially improve the quality of life of these patients and prevent some traumatic events, including a series of life-threatening accidents.

**Figure 1:**
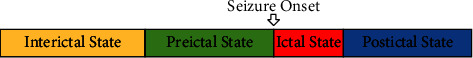
The sketch plot of four different states of epilepsy seizures.

Electroencephalography (EEG), as an electrophysiological monitoring approach to detecting brain electrical activity, has been proven to be a critical technique for diagnosing patients with epilepsy. Scalp EEG is typically noninvasive with multiple electrodes placed along the scalp [6]. It records the spontaneous electrical activity generated by brain neurons with high time resolution over a while. Scientists have found that it can be categorized into four different waveforms for scalp EEG records of patients with epilepsy. In the view of brain functional connectivity, these four different waveforms can be represented by four brain functional connectivity structures [7], corresponding to four different states of epilepsy seizures: (1) preictal state, which is the state before a seizure occurs; (2) ictal state, which is the onset state of seizure; (3) postictal state, which refers to the immediate state after a seizure; (4) interictal state, that is the state between postictal state and preictal state [8].  Figure 1 shows the sketch of the four states. Predicting seizures can be realized by detecting the preictal state, which can be achieved through discovering the changes in brain connectivity networks from interictal state to preictal state [9–12]. However, it is clinically difficult to identify the preictal state by visual inspection of scalp EEG signals to observe changes in the structure of brain connectivity networks. Therefore, powerful and explainable statistical methods are needed to determine the preictal state from scalp EEG recordings for seizure prediction.

Nowadays, brain functional connectivity modeling approaches have been proven to be a crucial tool in the neuroscience research field [12]. The brain can be seen as a complex network in which each brain region communicates and cooperates to carry out different functions [13]. However, the dysfunction in certain areas would interfere with the processing of upcoming information, consequently leading to network disorders and changes in a person's behavior [14]. Current research has shown that epilepsy is a specific disease related to the brain network abnormalities, and they also suggest that the brain functional connectivity of a particular patient would abnormal dynamic changes during seizures, and the forms of brain functional connectivity are different among four different states of epilepsy seizures [15]. Hence, it is reasonable and adequate to employ brain functional connectivity modeling approaches to predict seizures. Some previous studies have applied brain functional connectivity modeling methods to study epilepsy disease. For example, Williamson et al. [16] constructed multiple spatiotemporal correlation structure features from EEG data to classify the patients' preictal or interictal states. A potential limitation of this study is that it only used a cortical network rather than a whole-brain network and cannot extract all essential features, making it difficult to achieve excellent predictive performance. Varotto et al. [17] proposed a method that employed a partially directed coherence method to depict the brain functional connectivity network. However, this study did not use the brain network features to predict seizures. Furthermore, a more recent work [18] has proposed an automatic seizure prediction method based on a graph convolutional network. This method could achieve a good seizure prediction performance by exploring the critical brain network features. To the best of our knowledge, this method is an excellent approach for seizure prediction. However, there are two fatal issues with this method. First, since this method is based on the deep learning technique, the interpretability is relatively weak. Second, the algorithm of this method is too complicated to be applied by clinicians.

To address the issues mentioned above, we referred to the existing novel statistical analysis framework called simultaneous differential network analysis and classification for matrix-variate data (SDNCMV) [19] and based on the characteristics of scalp EEG data and epilepsy disease, we proposed an explainable statistical model for patient-specific seizure prediction. Before introducing the proposed method, we first briefly described the SDNCMV approach. This method was a two-stage data-driven approach that deals with fMRI data. The first stage estimated each subject's brain functional connectivity network and converted this network data into vector data for prediction in the next step. In the second stage, an ensemble prediction procedure was used to conduct the prediction results. In our study, we focused on using scalp EEG data to address patient-specific prediction problems. Since EEG data and fMRI data are in the same data format as matrix data [20, 21], we can refer to SDNCMV. However, there are still some differences between the scenarios of this study and those of Chen et al. [19] that used fMRI data to classify Alzheimer's disease. The fMRI data is a matrix data for each subject, while the EEG data is a matrix data for each epoch [22], which is artificially generated. More specifically, since the scalp EEG data used in this study have higher temporal resolution than fMRI data and the data epochs are small, we cannot use the SDNCMV method directly. We should assume that the brain functional connectivity for each subject is time-varying and then modify the first stage of SDNCMV to estimate the dynamic brain functional connectivity to obtain better performance. More details of our method will be introduced in the following section.

The significance of this study is that we proposed an explainable statistical method, which could be used to predict seizures based on the brain functional network. In addition, the proposed method is also computationally efficient, and the results can be easily interpreted. Therefore, this method can be better applied to the clinical and is more conducive to helping more patients with epilepsy.

## 2. Materials and Methods

### 2.1. Scalp EEG Data

The scalp EEG data used in this study are obtained from Children's Hospital Boston, the Massachusetts Institute of Technology (CHB-MIT) database [23] and Peking University First Hospital (FH-PKU) database.

The CHB-MIT database is available with open access at https://physionet.org/physiobank/database/chbmit/. The EEG recordings were collected from 23 children with intractable seizures, of which five males with age from 3 to 22, 17 females with age from 1.5 to 19, and one child with missing gender and age data. These recordings were grouped into 24 cases since the EEG data of patient ID chb21 was obtained one-half year after chb01 from the same child. The sampling frequency for this database was 256 Hz, and the international 10–20 EEG electrode positions and nomenclature system was used for these recordings. The difference between two adjacent electrodes obtained the signal measurement for each electrode in this scalp EEG data. In most of the 24 cases, there are 21 unique signals, while a few cases contain less or more. Hence, only the recordings that include these 21 unique signals were selected in this study to keep the results consistent.

The FH-PKU database is a private database containing 17 patients or cases. This scalp EEG data were recorded at a sampling rate of 500 Hz and used 19 signals in the international 10–20 system. In this database, a different method was used from the CHB-MIT database to measure the signal of each electrode. This method used the signal difference between the electrode and the fixed reference electrode. Furthermore, to ensure the accuracy of the results, the physiological state of the patients in different epochs was roughly the same.

In this study, we focus on patient-specific seizure prediction performance. For each patient in these two databases, the ictal state, the period when the patient experienced seizure onset, is easily detected from raw signals by doctors. Although the preictal state is challenging to identify and there is no gold standard, based on the ictal state, the preictal state can be defined by ourselves, which is the 30-minute window before a seizure occurs and is seen as the case state in this study. However, the interictal state, which is seen as the control state, is more difficult to define; it is hard to recognize the period of the postictal state. Hence, to eliminate the noise effect of the postictal state, recordings within 2 hours after the end of the seizure are removed. The period from this time to the next preictal state is defined as the interictal state. Moreover, if the time between two seizure periods is less than 2 hours, only the first one is selected for this study. We consider all epochs as samples in this study and divide the continuous EEG data within a preictal state and interictal state into nonoverlapping 60-second epochs. To reduce computational complexity, we average the data obtained every second for each signal. Then, the data of each epoch is in matrix form with 21 or 19 columns and 60 rows for the patients from CHB-MIT or FH-PKU database.

### 2.2. Dynamic Brain Functional Connectivity Estimation

This section introduces the procedure to estimate the individual-specific dynamic brain functional connectivity measures. For each individual, we denote **X**^*γ*^ ∈ *ℝ*^*p*×*q*^ and **Y**^*ϕ*^ ∈ *ℝ*^*p*×*q*^ as the raw scalp EEG data matrix of *γ*-th epoch for preictal state and *ϕ*-th epoch for the interictal state, respectively, where *p* represents the number of electrodes and *q* represents the number of time points. Based on the assumption that not every region of interest in our brain is connected, we estimate the dynamic brain functional connectivity within *γ*-th epoch for the preictal state and *ϕ*-th epoch for the interictal state via sparse precision matrices for **X**^*γ*^ and **Y**^*ϕ*^, which estimate the strength measures of brain connectivity via partial correlations. Here we mainly focus on the procedure of how to address the raw scalp EEG data matrix in the preictal state **X**^*γ*^, while **Y**^*ϕ*^ can be dealt with similarly.

Before introducing the detailed procedure, we follow the classical matrix normal distribution framework to define the distribution of **X**^*γ*^. Assume **X**^*γ*^ follows a matrix normal distribution for each *γ*, denoted as **X**^*γ*^ ~ *ℳ𝒩*(**M**_**X**_^*γ*^, Σ_**X**_*T*__^*γ*^ ⊗ Σ_**X**_*S*__^*γ*^), where Σ_**X**_*T*__^*γ*^=(Σ_**X**_*T*_,*ij*_^*γ*^) ∈ *ℝ*^*q*×*q*^ and Σ_**X**_*S*__^*γ*^=(Σ_**X**_*S*_,*ij*_^*γ*^) ∈ *ℝ*^*p*×*p*^ represent the covariance matrices of *p* electrodes locations and *q* time points for *γ*-th epoch, respectively. Then, for each time point *t* (1 ≤ *t* ≤ *q*) within the *γ*-th epoch, we have **X**_·*t*_^*γ*^ ~ *𝒩*(**M**_**X**,·*t*_^*γ*^, Σ_**X**_*S*__^*γ*^). If the brain functional network is stable within each epoch, there are lots of existing approaches to estimate the sparse precision matrix Ω_**X**_*S*__^*γ*^=(Σ_**X**_*S*__^*γ*^)^−1^ in the high-dimensional setting, such as Graphical Lasso [24] and CLIME [25]. However, in the current study of epilepsy, it is more reasonable to assume that the brain functional network is changing over time. Hence, we need to estimate the dynamic sparse precision matrix Ω_**X**_*S*__^*γ*^(*t*) for each time point based on time-varying covariance matrices, which can be achieved by

(1) $$\begin{array}{c} {\hat{\Omega }}_{X_{S}}^{\gamma }\left(t\right)=\underset{\Omega }{\arg\;\min}\left\{Tr\;\left({\hat{\Sigma }}_{X_{S}}^{\gamma }\left(t\right)\Omega \right)-\log\;\left|\Omega \right|+\lambda {\left\|\Omega \right\|}_{1}\right\}\text{,} \end{array}$$

where ${\hat{\Sigma }}_{X_{S}}^{\gamma }\left(t\right)=\underset{i}{\sum }w_{it}X_{\cdot i}^{\gamma }{\left(X_{\cdot i}^{\gamma }\right)}^{T}/\underset{i}{\sum }w_{it}$ is a weighted covariance matrix, and we adopt a symmetric non-negative kernel function *K*(·) to generate the over time weights as *w*_*it*_=*K*(|*i* − *t*|/*h*_*n*_). It is easy to find that this objective function is based on the Graphical Lasso and given the estimated ${\hat{\Sigma }}_{X_{S}}^{\gamma }\left(t\right)$, we can use the same algorithm to solve this optimization problem. Please refer to Graphical Lasso [24] for details. In practice, to obtain a better prediction performance, we substitute ${\hat{\Sigma }}_{X_{S}}^{\gamma }\left(t\right)$ by $\sum_{t=1}^{q}{\hat{\Sigma }}_{X_{S}}^{\gamma }\left(t\right)/q$, which is the average of ${\hat{\Sigma }}_{X_{S}}^{\gamma }\left(t\right)$ over *q* time points and then a unified sparse precision matrix estimation within each epoch, instead of multiple different sparse precision matrices ${\hat{\Omega }}_{X_{S}}^{\gamma }$, which can be used to estimate the brain functional connectivity measures. Although, here the sparse precision matrix is same for each time points within a specific epoch, it is generated via a time varying covariance matrix, so the brain functional network can be considered dynamic. In addition, we use Gaussian kernel function and set *h*_*n*_=*n*^1/3^ to calculate the weights *w*_*it*_ in the real application, where *n* is the sample size.

To sum, in this study, we adopt two symmetric matrices ${\hat{W}}_{X_{S}}^{\gamma }=\left({\hat{W}}_{X_{S},ij}^{\gamma }\right)\in {\mathbb{R}}^{p\times p}$ and ${\hat{W}}_{Y_{S}}^{\phi }=\left({\hat{W}}_{Y_{S},ij}^{\phi }\right)\in {\mathbb{R}}^{p\times p}$ to measure the dynamic brain functional connectivity strengths for *γ*-th epoch in preictal state and *ϕ*-th epoch in interictal state, respectively. Here, we vectorize them via extracting the upper triangular elements by row for each matrix and connecting them together, and define these vectors as $V_{X_{S}}^{\gamma }=Vec\left({\hat{W}}_{X_{S}}^{\gamma }\right)\in {\mathbb{R}}^{d}$ and $V_{Y_{S}}^{\phi }=Vec\left({\hat{W}}_{Y_{S}}^{\phi }\right)\in {\mathbb{R}}^{d}$ for each epoch in different states, in which each element represent an edge in brain functional network and the dimension *d* is equal to *p*(*p* − 1)/2. Assume there are *n*_1_ epochs in preictal state, *n*_2_ epochs in interictal state and totally *n*=*n*_1_+*n*_2_ epochs. Hence, we can define the predictor matrix used in predictive model as **V** ∈ *ℝ*^*n*×*p*(*p* − 1)/2^, where **V** can be expressed as the stack of matrix **V**_**X**_*S*__ ∈ *ℝ*^*n*_1_×*p*(*p* − 1)/2^ and matrix **V**_**Y**_*S*__ ∈ *ℝ*^*n*_2_×*p*(*p* − 1)/2^. In the following of this study, we are using **V** to serve as “Network Predictor Matrix.”

### 2.3. Dynamic Brain Functional Connectivity Estimation

Given the network predictor matrix, due to the complexity and high dimensionality of the data, we adopt a penalized and ensembled logistic regression method to predict seizures. For details, we use Lasso penalty to deal with high-dimension problem and bootstrap procedure to ensemble this penalized logistic regression. Let *Z* as the binary response variable and its observations are *Z*_1_,…, *Z*_*n*_, in which *Z*_*k*_=1(*k*=1,…, *n*_1_) means corresponding observations are from preictal state and *Z*_*k*_=0(*k*=*n*_1_+1,…, *n*) means corresponding observations are from the interictal state. We denote *P* as the probability of *Z*=1 and *B*(*b*=1,…, *B*) is the bootstrap times.

Now, we introduce this predictive method briefly, and for more details, please refer to Chen et al. [19]. We randomly sample *n*_1_ epochs in the preictal state and sample *n*_2_ epochs in the interictal state, respectively, with replacement. Then, we repeat the resampling *B* times, and for each time, we employ the high-dimensional logistic regression model. We define the *β*^(*b*)^ as the corresponding regression coefficients vector estimated by *b*-th model. If there is the coefficient in *β*^(*b*)^ which is not equal to 0, it indicates that the corresponding edge in the brain network is meaningful for distinguishing between the preictal state and the interictal state. Finally, after whole resampling procedure, we define the estimated coefficients vector as ${\hat{\beta }}^{\left(b\right)}$(*b*=1,…, *B*) and outcome for test sample as ${\hat{P}}^{\left(b\right)}$. Hence, we use ${\hat{P}}_{B}={\hat{P}}^{\left(b\right)}/B$ to denote the proportion of a new epoch is assigned to preictal state and $\psi_{i}=1/B\sum_{b=1}^{B}I\left({\hat{\beta }}_{i}^{\left(b\right)}\neq 0\right)$ to denote the weight for corresponding edge in the brain network. The greater the weight, the more important this edge is. This demonstrates the interpretability of our method.

### 2.4. Performance Evaluation Measures

The evaluation measures that we adopt for the performance of seizure prediction are Sensitivity Rate (SENS), False Discovery Rate (FDR), and Area Under Curve (AUC). The SENS measures the proportion of epochs from the preictal state with a positive result, and FDR is defined as the proportion of all epochs predicted from the preictal state, which is not. Since the values of these two criteria change with the cutoff value, we select the cutoff with the highest prediction accuracy. To avoid different cutoffs affecting performance, we also present the AUC values, which is a cutoff-independent measure and would be the most comprehensive measure.

## 3. Results

This section illustrates the results of the proposed method in seizure prediction by applying it to the CHB-MIT and FH-PKU databases.

Table 1 presents the seizure prediction results for 24 patients in the CHB- MIT database. To prove that features derived via a dynamic brain functional network contain more information and improve prediction accuracy, we convert the raw scalp EEG data matrix for each epoch to a vector and stack these *n* vectors into a data matrix of dimension *n* × *pq*. Then, we feed this data matrix into our ensemble prediction model for comparison. Furthermore, considering that the raw data may contain a small amount of information, we also combine the predictor matrix derived from brain network data (BN Data) and the predictor matrix derived from raw scalp EEG data (Raw Data) as a predictor to observe its prediction performance. The results in Table 1 show that satisfactory prediction results can be obtained by using network features, while the prediction results obtained via using raw data as input are no different from random guessing. In addition, using the combination of brain network data and raw EEG data can also achieve satisfactory prediction. However, it is still worse than using brain network data only. This is because the raw data cannot provide any valuable information for prediction, so increasing these redundant variables from raw data makes prediction performance worse.

The seizure prediction results for 17 patients from the FH-PKU database are presented in Table 2. As done in Table 1, we compare the prediction performance of our method using three kinds of input features. In Table 2, we show that network features can also achieve accurate predictions. Although the prediction result obtained using the raw data is higher than random guessing, it is still unsatisfactory. Furthermore, unlike the CHB-MIT data, in this dataset, since the raw data can provide some valuable information for prediction, it is found that the best prediction performance can be obtained using the combined data.

## 4. Discussion

In this study, we have proposed an explainable statistical method to predict epileptic seizures, which is helpful in raising the alarm before seizures. More concretely, our method uses scalp EEG data to construct a dynamic brain functional connectivity network via a time-varying precision matrix estimation approach. Then, we treat these brain functional connectivity measures as predictor variables for the ensembled prediction model. Finally, through the proposed method in this study, we can obtain accurate prediction results by using these electrodes with overactive electrical discharges as predictors. In the following, we would like to discuss the findings of this study, and at the end, we will present some future research directions.

### 4.1. Relationship to Other Studies

Our study is not the first to use scalp EEG data to extract brain connectivity signatures for seizure prediction. There have been lots of state-of- the-art approaches, such as Gemein et al. [26], Tsiouris et al. [27], and Truong et al. [28]. So far as we know, the method called STS-HGCN-AL proposed by Yang et al. [18] can achieve a better seizure prediction performance among these approaches. We speculate that if our method outperforms STS- HGCN-AL, our method will outperform all existing methods. Hence, in this study, we only compare the prediction performance of our method with the method STS-HGCN-AL in the public database CHB-MIT database. It should be noted that it is difficult to draw a direct comparison due to different data preprocessing, such as how to choose the length of the epoch, when the preictal state starts, etc. In addition, the method STS-HGCN-AL has more strict requirements for the raw data, so this method can address part of patients' data within the CHB-MIT database, while our method can deal with all patients' data in terms of seizure prediction. We choose the AUC value as a measure of prediction performance to compare these two methods for the part of patients' data. From the results in Table 1, it is not difficult to see that our method can predict more patients and has a higher AUC value of 94% in these patient data, which can suggest that our method performs better than the existing method STS-HGCN-AL in terms of predicting epileptic seizures.

**Figure 2:**
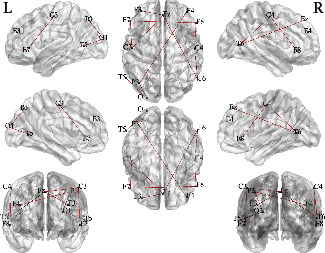
Top 10 brain functional connections that affect seizures for patient 210486.

**Figure 3:**
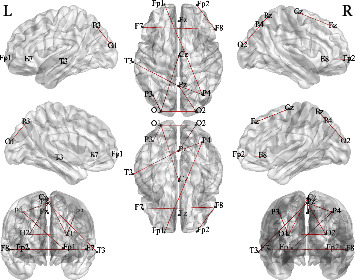
Top 10 brain functional connections that affect seizures for patient 210494.

Furthermore, since our method uses brain functional connectivity features to predict seizures, in this study, in addition to the prediction performance, we also briefly discuss the performance of critical feature selection. We randomly select two patients from the FH-PKU database, the patient 210486 and patient 210494. Based on the order of the derived weights *ψ* for each edge in the brain network, we show the top 10 critical brain functional connectivity features identified by our model in Table 3 and Figures 2 and 3. Then, based on the weights for each edge in Table 3, we may consider the features with larger weights as identifying potential connectome biomarkers. For the identification of potential connectome biomarkers, there are many existing methods, such as Song et al. [29], Lu et al. [30], and Ding et al. [31]. However, there is no gold standard for the public datasets identifying potential connectome biomarkers. We cannot prove the effectiveness of our method for this issue and thereby cannot compare our method with existing methods. We just put the identification results here without evaluating the performance, and for this issue, we will leave it as a direction for future research.

### 4.2. Limitations and Future Directions

Although our method achieves satisfactory results in terms of seizure prediction, it still leaves much space for improvement to obtain even more realistic models. The limitations of our method proposed in this study mainly concentrated on three aspects. At first, for the sake of simplicity of our model, we have assumed that the raw scalp EEG data of each patient comes from a normal distribution. However, in the real world, we cannot know the actual distribution of the raw data. Hence, we are currently applying some statistical methods to relax the normal assumption for this problem. Secondly, in our model, we have used the same time window to define the preictal state of each patient, while in the real world, each patient has its heterogeneity and the time window of the preictal state is diverse. Hence, we plan to focus on how to estimate an optimal time window of the preictal state in the future. In the end, the study only briefly discussed how to identify potential connectome biomarkers using our method but did not test whether these biomarkers actually affected epilepsy. Therefore, in the future, we will conduct hypothesis tests in this field to demonstrate the efficiency and accuracy of our method in identifying potential connectome biomarkers.

### 4.3. Potential Applications of the Method in Treatment of Epilepsy

Epileptic seizures are sudden and have no apparent signs. The prediction of epileptic seizures can significantly enhance the effect of epilepsy treatment, improve the quality of life of patients with epilepsy, and reduce the mortality due to epileptic seizures, so the accurate prediction of epileptic seizures in the clinical application has a vital significance. Our experimental results and the comparison with previous work demonstrate that the proposed method is efficient. This gives the patient enough time to take action to cope with the seizure and reduce anxiety and trauma.

Patients with epilepsy after regular ASMs treatment, there is still one-third of patients with epilepsy that cannot be controlled. Uncontrolled seizures have severe impacts on patients' cognition, memory, quality of life, social psychology, and the growth and development of children. In recent years, imaging, electroencephalography, genetics, and other diagnostic techniques have been continuously improved, and the efficacy and safety of surgical resection have been recognized. For patients with drug-resistant epilepsy with a clear epileptogenic focus and a low surgical risk, surgical resection should be considered as soon as possible. The accuracy of the connectome biomarker identification may help to determine the epileptic region before epilepsy surgery, which is the key to ensuring the success of epilepsy surgery. Hence, finding the right target remains the essential prerequisite for our new drug development, and accurate connectome biomarker identification can provide potential therapeutic targets. In this study, we have briefly discussed that our method may enable potential connectome biomarker identification, which is essential for physicians to conduct the preoperative evaluation and develop new drugs or treatments for epilepsy, but this needs to be further validated in the future.

## 5. Consent

The parents of the patients signed written informed consent and agreed with their children's participation in this study and allowing the use of the relevant data and information for scientific research.

## Figures and Tables

**Table 1:** Seizures prediction results for the patients in CHB-MIT database via our method and STS-HGCN-AL method.

Patient ID	BN data			Raw data			BN + raw data			STS-HGCN-AL		
	AUC	SENS	FDR	AUC	SENS	FDR	AUC	SENS	FDR	AUC	SENS	FDR
chb01	0.968	95.0	0.060	0.496	47.5	0.374	0.970	95.0	0.088	0.996	100	0.000
chb02	0.984	100	0.102	0.634	75.0	0.473	0.973	90.0	0.059	0.897	100	0.145
chb03	0.992	95.5	0.022	0.691	50.0	0.193	0.989	95.5	0.029	0.928	83.3	0.173
chb04	0.867	86.7	0.290	0.595	50.0	0.247	0.825	93.3	0.362	NA	NA	NA
chb05	0.835	91.5	0.173	0.458	100	0.946	0.778	60.0	0.135	0.875	100	0.000
chb06	0.804	84.3	0.344	0.505	22.9	0.154	0.779	65.7	0.205	0.906	100	0.162
chb07	0.978	93.3	0.067	0.583	43.3	0.221	0.975	93.3	0.057	NA	NA	NA
chb08	0.953	85.0	0.078	0.548	40.0	0.200	0.933	85.0	0.100	0.999	100	0.000
chb09	0.963	90.0	0.043	0.553	96.7	0.815	0.950	90.0	0.069	0.843	100	0.092
chb10	0.947	86.7	0.073	0.544	25.0	0.110	0.922	85.0	0.127	0.977	83.3	0.171
chb11	0.990	95.0	0.039	0.696	80.0	0.459	0.989	95.0	0.036	0.940	100	0.123
chb12	0.911	76.8	0.095	0.479	96.4	0.919	0.904	87.5	0.162	NA	NA	NA
chb13	0.998	96.3	0.000	0.506	22.2	0.100	0.996	96.3	0.025	0.915	85.7	0.109
chb14	0.872	95.0	0.015	0.490	42.5	0.335	0.826	92.5	0.369	0.976	100	0.104
chb15	0.836	99.8	0.231	0.433	84.6	0.838	0.793	73.6	0.306	NA	NA	NA
chb16	0.950	93.3	0.189	0.486	40.0	0.322	0.904	96.7	0.211	0.954	87.5	0.187
chb17	0.960	95.0	0.080	0.542	75.0	0.587	0.954	90.0	0.073	0.826	100	0.237
chb18	0.912	100	0.300	0.553	30.0	0.143	0.901	87.5	0.259	0.992	75.0	0.138
chb19	0.976	91.0	0.046	0.610	63.6	0.390	0.974	100	0.167	0.991	100	0.038
chb20	0.987	100	0.077	0.386	33.3	0.319	0.980	100	0.117	0.982	100	0.184
chb21	0.957	90.0	0.085	0.451	5.0	0.002	0.923	85.0	0.115	0.833	100	0.156
chb22	0.924	96.3	0.245	0.485	40.7	0.283	0.860	85.2	0.219	0.997	100	0.000
chb23	0.994	100	0.011	0.409	10.0	0.043	0.990	100	0.031	0.990	100	0.047
chb24	1.000	100	0.000	0.599	87.0	0.637	1.000	100	0.000	NA	NA	NA
Average	0.940	93.0	0.111	0.530	52.5	0.380	0.920	89.3	0.138	0.938	95.5	0.109

**Table 2:** Seizures prediction results for the patients in FH-PKU database via our method.

Patient ID	BN data			Raw data			BN + raw data		
	AUC	SENS	FDR	AUC	SENS	FDR	AUC	SENS	FDR
200002	0.950	100	0.125	0.637	40.0	0.025	0.945	80.0	0.000
210416	0.991	95.0	0.026	0.945	100	0.184	0.983	95.0	0.053
210443	0.957	90.0	0.000	0.726	70.0	0.359	0.967	90.0	0.000
210447	0.900	89.5	0.237	0.749	78.9	0.342	0.865	94.7	0.316
210454	0.891	85.0	0.105	0.834	100	0.447	0.930	90.0	0.132
210460	0.996	100	0.071	0.847	90.0	0.284	0.995	96.7	0.035
210465	0.992	95.0	0.035	0.790	80.0	0.319	0.985	100	0.113
210467	0.939	90.0	0.029	0.795	70.0	0.143	0.967	90.0	0.029
210470	0.975	90.0	0.000	0.820	100	0.447	0.978	90.0	0.000
210471	0.929	80.0	0.025	0.579	50.0	0.256	0.910	85.0	0.154
210477	0.971	87.5	0.026	0.651	95.8	0.692	0.927	87.5	0.103
210486	0.995	100	0.025	0.869	65.0	0.026	0.989	95.0	0.051
210489	0.978	90.0	0.026	0.749	85.0	0.368	0.988	90.0	0.026
210494	1.000	100	0.000	0.949	92.9	0.154	1.000	100	0.000
210498	0.992	100	0.028	0.611	70.0	0.451	0.987	100	0.056
210499	0.948	80.0	0.013	0.730	60.0	0.197	0.970	100	0.158
210503	0.877	80.0	0.040	0.981	100	0.067	0.986	100	0.080
Average	0.958	91.3	0.048	0.780	79.3	0.280	0.963	93.1	0.077

**Table 3:** Epileptogenic focus localization results for the two patients in FH-PKU database.

	210486	*ψ*	210494	*ψ*
1	Occipital L ↔ temporal L	1.00	Parietal L ↔ occipital L	1.00
2	Frontal L ↔ frontal R	0.91	Occipital L ↔ frontal M	0.99
3	Frontal R ↔ parietal L	0.91	Parietal R ↔ parietal M	0.89
4	Parietal R ↔ temporal R	0.75	Frontal M ↔ frontal M	0.66
5	Parietal L ↔ occipital L	0.69	Frontal L ↔ parietal R	0.65
6	Temporal L ↔ frontal M	0.66	Temporal L ↔ parietal M	0.56
7	Parietal L ↔ frontal L	0.65	Occipital R ↔ parietal M	0.56
8	Parietal L ↔ frontal M	0.65	Frontal R ↔ frontal R	0.54
9	Parietal R ↔ frontal R	0.56	Occipital L ↔ occipital R	0.51
10	Frontal L ↔ frontal M	0.55	Frontal L ↔ frontal R	0.50

## Data Availability

The original contributions presented in this study are included in the article; further inquiries can be directed to the corresponding authors.
